# The Spanish Military Medical Service in Cuba

**Published:** 1898-07

**Authors:** 


### THE SPANISH MILITARY MEDICAL SERVICE IN CUBA.

We can afford to be generous enough not to let our natural feeling against Spain at the present juncture blind our eyes to the one aspect of the Spanish occupation of Cuba which is extra-national—the services of the Spanish military medical officers—and take credit on behalf of the only cosmopolitan profession for their record as given by a special correspondent in the *Lancet* for June 11th. The writer thus closes an exhaustive report: "Altogether, and for the entire medical military services of Cuba from the commencement of the insurrection to the middle of the year 1897, no less than six hundred surgeons have been engaged in attending to the sick and wounded. Of these, fifty have died in the accomplishment of their duty, the greater number being victims of yellow fever. Six dispensing chemists have likewise met with the same honorable fate. Twenty-five surgeons were wounded on the field of battle and four were killed. There can be no doubt that the military medical staff showed under the most trying and dangerous circumstances all the courage and heroism which have so often distinguished the Spanish people in general and the medical profession in particular." While patriotism is limited by geographical confines, we should be proud as a profession to feel that professional zeal and loyalty are universal in their reach, and that the ever-earnest contribution of even the humblest member affords an increment to the treasury of humanity.—*New York Medical Journal.*

